# A 10-year follow-up study of completers versus dropouts following treatment with an integrated cognitive-behavioral group therapy for eating disorders

**DOI:** 10.1186/s40337-017-0182-y

**Published:** 2017-11-17

**Authors:** Yuri Okamoto, Yoshie Miyake, Ichie Nagasawa, Kazuhiro Shishida

**Affiliations:** 1 Health Service Center, Hiroshima University, 1-7-1, Kagamiyama, Higashihiroshima, Hiroshima, 739-8514 Japan; 2 Department of Psychiatry and Neurosciences, Hiroshima University, 1-2-3, Kasumi, Minami-ku, Hiroshima, 734-8551 Japan

**Keywords:** Eating disorder, Cognitive-behavioral therapy, Group therapy, Prognosis, Depressive symptoms, Coping, Self-esteem, Dropout

## Abstract

**Background:**

Cognitive-behavioral therapy (CBT) has been recommended for the treatment of eating disorders, and group therapy is known to have certain advantages over individual therapy. The aim of the current study was to compare the 10-year prognosis of patients who completed integrated group CBT with those who dropped out and to examine the effect of completion of group CBT on the prognosis.

**Methods:**

The participants were 65 adult patients with eating disorders. All patients were women and Japanese. The average age (19–37) of the patients was 25.1 ± 3.8 years, and the average body mass index (BMI) was 17.7 ± 2.0. We conducted integrated group CBT with the patients and compared eating disorder symptoms, mood states, coping styles, and self-esteem before and after therapy. Furthermore, we compared clinical features and the 10-year prognosis of patients who completed the treatment and those who dropped out.

**Results:**

After 10 sessions of group therapy, Eating Attitudes Test scores, Profile of Mood States depression scores, and Coping Inventory for Stressful Situations emotion-oriented scores decreased, while Rosenberg’s Self-Esteem Scale scores increased. Regarding the results of the 10-year follow up, the completer group had more patients with a good prognosis. In contrast, the dropout group had more patients with a poor prognosis.

**Conclusions:**

Those who completed the integrated group CBT had a good prognosis. Group therapy gives the patients an opportunity to form peer relationships, and helps them to develop communication and socialization skills. Furthermore, in the group therapy sessions, the patients develop self-awareness by listening to other members of the group and they also develop interpersonal relationships. This effect may be temporary, but experience of group therapy may provide hope for the patient and increase the chance of the patient continuing treatment.

**Trial registration:**

Retrospectively registered in University Hospital Medical Information Network in Japan: No. 000028868 (May 19th, 2017).

## Plain English summary

We compared the 10-year prognosis of patients who completed integrated group CBT with those who dropped out and examined the effect of completing group CBT on prognosis. After group CBT, eating attitudes and depressive symptoms decreased, and self-esteem increased significantly. We also compared the clinical features and 10-year prognosis of patients who completed the study and those who dropped out. The completer group had a significantly higher number of patients who had a good outcome, and the dropout group had a significantly higher number of patients who had a poor outcome. The integrated group CBT experience might have positive long-term effects.

## Background

Eating disorders are serious illnesses that can be difficult to treat [1]. Many serious problems are associated with eating disorders [2, 3], such as 1) significant mortality rates [4], 2) suicidal behavior [5, 6], 3) high medical costs [7], 4) high rates of comorbidity [8], and 5) high association with childhood trauma, such as abuse. Nonetheless, the existing evidence-based treatments are still limited in their effectiveness.

In the National Institute for Health and Clinical Excellence guidelines [9], cognitive behavioral therapy (CBT) is recommended for anorexia nervosa (AN), bulimia nervosa (BN), and binge eating disorder (BED). Fairburn [10] reported that most eating disorders have a mixed clinical presentation, in which the features of AN, BN, BED, and those of incomplete types overlap in various ways. Fairburn adapted CBT to make it transdiagnostic, i.e., appropriate all eating disorder psychopathologies, and reported that this Enhanced CBT (CBT-E) outcome did not depend on the type of eating disorder involved [11]. Accordingly, we conducted CBT in a transdiagnostic sample.

We conducted CBT in a group setting for the following two reasons. First, group therapy is time-efficient. In Japan, outpatient clinics treat many patients and it is not possible to commit large amounts of time to individual treatment. With group therapy, time problems can be solved to an extent. In addition, group therapy reduces patient payment to 54–70% of that of individual psychotherapy; therefore, group therapy is cost-effective. Many reports have indicated that group therapy for eating disorders is cost-effective and cost-beneficial [12–14]. Moreover, both CBT and interpersonal therapy have demonstrated good outcomes [15]. The second reason is that group psychodynamics work therapeutically. Group therapy gives patients the opportunity to be supported by others and to support others. Yalom et al. [16, 17] reported that interpersonal learning is an important and dynamic factor in group-based psychotherapy that allows group members to share aspects of their emotions, thoughts, and perceptions while receiving feedback from fellow group members in a safe and collaborative setting. Because recovery from eating disorders takes considerable time, many patients lose motivation during treatment. We believe that group therapy can contribute to enhancing and continuing motivation for treatment as the patients support each other.

In this study, it was difficult to apply Fairburn’s CBT-E in its entirety because in group therapy, it is difficult to carefully intervene in patient’s cognitions as is done on a one-to-one basis in individual therapy. Therefore, we partially adopted CBT-E and added a variety of approaches for conducting group therapy that motivate recovery efforts. In the group CBT, a self-assertion session seemed necessary. This is because Japanese individuals, and especially those with eating disorders, are not typically self-assertive, and it is important for patients to address this in order to have acceptable experiences within the group. A Japanese study reported that a combination program of CBT with assertive training and self-esteem enhancement was effective [18]. In addition, we considered problem-solving techniques important. Patients with eating disorders feel pressured by numerous problems, isolated, and helpless. Clarifying problems and considering solutions within the group setting helps alleviate such feelings. Pettersen [19] reported that recovery from eating disorders included improved acceptance of oneself, interpersonal relations, problem solving, and body satisfaction, which were not entirely dependent on symptom absence.

In contrast, eating disorders may be resistant to change, and dropout rates from treatment are high [20, 21]. A review of 19 studies reported that the therapeutic alliance might be an important factor in eating disorder treatment [22]. Another systematic review emphasized the importance of early-stage motivation for change as a predictor of treatment outcome [23]. A 2-year follow-up study reported that temperament is related to dropout and one of the reasons for dropout is lack of motivation [24]. Therefore, motivation for change is important to prevent dropout from treatment; we believed that integrated group CBT might help support motivation for change. We hypothesized that an integrated program incorporating CBT might have a positive impact on continued treatment and prognosis, even for short-term interventions.

The purpose of the current study was to compare the 10-year prognosis of patients who completed integrated group CBT with those who dropped out and to examine the benefits of completing the group CBT.

## Methods

### Procedure

Participants were recruited from the outpatient clinic of the Department Psychiatry, Hiroshima University Hospital from 1995 to 2005. We excluded patients under 18 years of age, those with psychotic disorders, intellectual disability, personality disorder with self-destructive behavior, or those at high risk of suicide. We recruited patients whose treatment had started within the past year.

We interviewed each patient in advance, judged whether the patient did not fit the exclusion criteria, and introduced the research to the subjects. For the patients from whom consent was obtained, group therapy was conducted for 6 or 7 patients in the order of registration. Psychological measurements were taken before and after group therapy. We performed group therapy in parallel with outpatient consultation, but did not allow concurrent individual psychotherapy or nutritional counseling. However, 15 patients had received nutritional counseling before the group therapy started. Regular outpatient treatment consisted of a medical consultation that included simple counseling for about 15 min in the outpatient clinic and medication.

Those who completed group therapy with fewer than 3 absences were regarded as completers, and those who were absent 5 times or more were regarded as dropouts. All participants fell into one of these two groups. We investigated the prognosis after 1, 5, and 10 years after group therapy of completers and dropouts who we were able to follow up. We conducted the following assessments after 5 and 10 years: BMI, number of regular meals/day, instances of overeating (number of times and extent of overeating), frequency of vomiting, use of laxatives or diuretics, excessive exercise, alcohol and tobacco use, self-injurious behavior, other problem behaviors, comorbidities, symptoms such as depression and anxiety, interpersonal relationship situation, marriage and childbirth, work, and social activities. Based on these items, we evaluated the prognosis as described below.

#### Evaluation of prognosis

We used improvements in eating behavior and social adjustment as parameters for the prognostic evaluation. The conditions for determining improvements in eating behavior were: three meals per day were regularly eaten, the frequency of overeating was less than once a week, the BMI was 17.5 or more, and the behaviors were sustained for more than 1 year. Improvements in social adaptation were judged using the Global Assessment of Functioning (GAF). GAF is a component of the DSM-IV, not the DSM-5. However, GAF has been widely used and it is easy to administer. We considered a score of higher than 80 on the GAF to be an indicator of good social adaptation. We considered the patients whose eating disorder symptoms improved and who had adapted well to society as having made “good progress,” the patients whose symptoms did not change as having “no change,” and the patients whose symptoms got worse or were maladaptive (GAF score less than 50) as having “poor progress.”

This study received approval of Hiroshima University Medical Ethics Committee (No: E776). In addition, we retrospectively registered the study in University Hospital Medical Information Network in Japan (No.000028868, May 19th, 2017).

### Participants

The participants were 65 adult patients with eating disorders diagnosed in accordance with the Diagnostic and Statistical Manual of Mental Disorders (DSM), 4th edition (DSM-IV) and re-diagnosed with DSM 5th edition (DSM-5) based on the medical records and discussion with the attending doctor. However, although The Global Assessment of Function (GAF) is not present in the DSM-IV, we used GAF because it is convenient. All patients were Japanese women. The average age (19–37) of the patients was 25.1 ± 3.8 years, age of onset was 17.8 ± 2.8 years, duration of illness was 6.8 ± 4.2 years, and the average body mass index (BMI) was 17.7 ± 2.0 kg/m^2^. The included cases were 18 cases of AN restricting type (AN-R), 12 cases of AN binge-eating/purging type (AN-BP), 24 cases of BN, 9 cases of BED, and 2 cases of other specified feeding or eating disorder (OSFED). According to comorbidities, 13 cases were of depressive disorder, 10 of panic disorder, 3 of obsessive-compulsive disorder, and 11 were of personality disorder (7 borderline personality disorder and 4 schizoid personality disorder).

Forty-eight patients completed the therapy sessions (they attended more than 8 sessions), and 17 patients (26.1%) dropped out of the study. Of the 17 patients, 3 dropped out initially, 8 within 3 weeks (3rd session) of the first session, and 6 within 1 month (4–5th session).

### Intervention

The structure of the group therapy was as follows: 1) closed membership, 2) group size limited to no more than eight members, 3) weekly meetings (90 min, 10 sessions), and 4) 2–3 staff members (2 psychiatrists usually participated, sometimes a psychologist joined) present.

#### Session 1, 2: “What is an eating disorder?”

In these sessions, we used a psycho-educational approach. In addition, we adopted Fairburn’s “formulation” and self-monitoring. We created the “formulation” and learned about self-monitoring through real time meal, situation, and mood records. The participants were encouraged to perform real-time self-monitoring as homework, and in each session after that, we analyzed the records objectively and discussed countermeasures.

#### Session 3: “Thinking about body image and evaluating symptoms”

In this session, we evaluated body image and body checking behavior. We assessed the extent to which the patients were preoccupied by their weight.

#### Session 4, 5: “Tackling difficult problems”

In these sessions, we clarified the various general problems, especially interpersonal problems the patients faced, and discussed new ways of coping with the problems. The purpose of these sessions was to improve the coping skills of the patients.

#### Session 6, 7: “Asserting myself”

In these sessions, we discussed the scenarios in which the patients experienced difficulties in self-assertiveness, and trained the patients with regard to self-assertiveness through role-play. The purpose of the sessions was to improve the skills the patients needed to assert themselves.

#### Session 8, 9: “Thinking about cognition”

In these sessions, the patients learned about 1) common cognitive styles in eating disorders including working to reconsider the “formulation” created in the first session, 2) using a thought record list to examine their cognitive style, and 3) examining their perception of their weight and body shape, and their views of their interpersonal relationships.

#### Session 10: “If you recovered from your eating disorder?”

In this session, we encouraged the patients to imagine themselves as someone who recovered, discuss the merits and demerits of recovery, and uncover factors that may hinder their recovery. The purpose of this session was to reinforce the motivation to continue treatment.

In the last intervention, we directed the patients to continue using the content of the session by themselves, and to report the progress to their doctor at the time of consultation.

### Measures

#### Eating attitudes Test-26 (EAT-26)

The Eating Attitudes Test-26 (EAT-26) is a 26-item self-reported measure of eating attitudes. The original scale consists of 40 items (EAT-40) but we used Garner’s modification, namely the shorter EAT-26. Garner reported that the EAT-26 was highly correlated with the EAT-40, and that the EAT-26 was a reliable, valid, and economical instrument [25]. It is scored using a six-point Likert-type scale, with options ranging from “not at all” to “extremely.” We used the Japanese version translated by Baba et al. in 1993. Cronbach’s alpha coefficient scores were 0.85–0.94 [25, 26].

#### Profile of mood states (POMS)

The Profile of Mood States (POMS) was developed by McNair et al. [27], and is administered to patients to assess their mood states. It is a 65-item self-report measure of six subscales comprising tension/anxiety, depression/dejection, anger/hostility, vigor/activity, fatigue/inertia, and confusion/bewilderment. It is scored using a five-point Likert-type scale, with options ranging from “not at all” to “extremely.” We used the Japanese version translated by Yokoyama et al. in 1994. Cronbach’s alpha coefficient scores were 0.76–0.95 [27, 28].

#### Coping inventory for stressful situations (CISS)

The Coping Inventory for Stressful Situations (CISS) was developed by Endler et al. [29]. It is a 48-item self-report measure that is scored using a five-point Likert-type scale, with options ranging from “not at all” to “extremely.” This scale evaluates three coping behavior patterns as follows: task-oriented, emotional-oriented, and avoidance-oriented coping. Task-oriented coping is considered to be an adaptive coping behavior; in contrast, emotion-oriented coping is considered to be a non-adaptive coping behavior, which includes self-criticism and anger. We used the Japanese version translated by Furukawa et al. in 1993. Cronbach’s alpha coefficient scores were 0.75–0.89 [30].

#### Rosenberg’s self-esteem scale (RSES)

Rosenberg’s Self Esteem Scale (RSES) was developed by Rosenberg [31]. It is a 10-item self-report measure that is scored on a four-point Likert-type scale, with options ranging from “not at all” to “extremely.” We used the Japanese version translated by Yamamoto et al. in 1982. Cronbach’s alpha coefficient scores were 0.82–0.86 [32].

### Statistical analysis

We used IBM SPSS version 21 statistics for statistical analysis. We used paired t-tests to analyze the self-rating scales before and after intervention, Mann-Whitney U-tests to compare the self-rating scales between patients who completed therapy and those who dropped out of the study, and chi-square tests for comparing the frequencies of good outcome, no change, and poor outcome. We assumed *p* < 0.01 indicated significant differences.

## Results

### Comparison of patients who completed therapy and those who dropped out

The results of the comparison of the patients who completed the integrated CBT group therapy session and those who dropped out of the study are shown in Table 1. The distribution of the types of eating disorder, age, BMI, and comorbid personality disorders did not differ between the two groups. The dropout group had a significantly higher number of patients who dropped out from outpatient consultation than the completers group (χ^2^(1) = 14.6, *p* < 0.01). The POMS, tension/anxiety scores, and anger/hostility scores of the dropout group were tend to be higher than those of the completer group (*p* < 0.05).

**Table 1:** Comparison between completers and dropouts (1)

	Completers (*N* = 48)	Dropouts (*N* = 17)	*p*
Subtype	AN-R 1,AN-BP BN 18,BED 7, OSFED 2	AN-R 5,AN-BP 4 BN 6,BED 2	
Age	25.1 ± 3.8	25.2 ± 6.2	NS
Age of onset	17.9 ± 2.7	17.7 ± 3.0	NS
Duration of illness	6.7 ± 4.2	5.5 ± 3.8	NS
BMI	17.7 ± 2.0	18.5 ± 2.8	NS
Education			
high school graduate	27 (56.3%)	11 (64.6%)	NS
college student	4 (8.3%)	1 (5.9%)	NS
college graduate	17 (35.4%)	6 (3.5%)	NS
Marital status (married)	11 (22.9%)	4 (23.5%)	NS
Comorbidities			
depressive disorder	9 (18.8%)	4 (23.5%)	NS
panic disorder	7 (14.6%)	3 (17.6%)	NS
obsessive-compulsive	2 (4.1%)	1 (5.9%)	NS
personality disorder	7 (14.6%)	4 (23.5%)	NS
Dropout from outpatient consultation	2 (4.2%)	8 (47.1%)*	<0.01
EAT-26	27.2 ± 7.5	27.3 ± 8.4	NS
POMS			
Tension-anxiety	21.4 ± 7.0	25.6 ± 5.6	0.029
Depression	30.9 ± 10.6	32.2 ± 8.6	NS
Anger-hostility	18.3 ± 9.7	23.7 ± 8.3	0.044
Vigor-activity	12.9 ± 3.9	14.6 ± 3.5	NS
Fatigue	18.1 ± 5.0	16.9 ± 3.2	NS
Confusion-bewilderment	16.4 ± 5.3	8.6 ± 5.2	NS
CISS			
Task-oriented coping	53.2 ± 8.0	50.0 ± 6.5	NS
Emotion-oriented coping	60.6 ± 7.0	60.5 ± 7.4	NS
Avoidance-oriented coping	50.9 ± 5.1	52.0 ± 6.2	NS
RSES	17.9 ± 5.7	18.6 ± 3.8	NS

*AN-R* anorexia nervosa restricting type, *AN-BP* anorexia nervosa binge eating/purging type, *BN* bulimia nervosa, *BED* bing- eating disorder, *OSFED* other specified feeding or eating disorder, *EAT-26* Eating Attitudes Test-26, *POMS* Profile of Mood Status, *CISS* Coping Inventory for Stressful Situation, *RSES* Rosenberg’s Self-Esteem Scale

*There were significantly more dropout cases from outpatient consultation. Tension-anxiety and anger-hostility of POMS in the dropouts group were tend to be higher than those of completer group

### Changes before and after group therapy

The changes in the EAT, POMS, CISS, and RSES scores are shown in Table 2. After group therapy, the EAT scores and the depression scores on the POMS decreased (p < 0.05). The emotion-oriented scores on the CISS decreased and the RSES scores increased (*p* < 0.05) after group therapy. In relation to stress coping, emotion-oriented coping was reduced after the intervention. Emotion-oriented coping is considered to be a non-adaptive coping behavior.

**Table 2:** Assessments before and after group therapy

		before	after	*p*
EAT-26		27.2 ± 7.5	25.0 ± 6.8	0.043
POMS	Tension-Anxiety	21.4 ± 7.0	19.7 ± 4.8	NS
	Depression	30.9 ± 10.6	27.4 ± 9.5	0.044
	Anger-Hostility	18.3 ± 9.7	17.5 ± 6.3	NS
	Vigor-activity	12.9 ± 3.9	13.0 ± 3.5	NS
	Fatigue	18.1 ± 5.0	16.5 ± 5.4	NS
	Confusion-bewilderment	16.4 ± 5.3	16.4 ± 4.5	NS
CISS	Task-oriented coping	53.2 ± 8.0	54.6 ± 7.4	NS
	Emotion-oriented coping	60.6 ± 7.0	58.4 ± 5.9	0.044
	Avoidance-oriented coping	50.9 ± 5.1	50.7 ± 5.6	NS
RSES		17.9 ± 5.7	19.3 ± 3.7	0.044

*EAT-26* Eating Attitudes Test-26, *POMS* Profile of Mood Status, *CISS* Coping Inventory for Stressful Situation, *RSES* Rosenberg’s Self-Esteem Scale

EAT-26, Depression of POMS, and Emotion-oriented coping of CISS were tend to decreased and RSES was tend to increased after group therapy

### Ten-year outcomes of those who completed the study and those who dropped out

Table 3 shows the 10-year outcomes of 36 patients (75% of original sample) who completed the therapy sessions and 10 patients (58.8% of original sample) who dropped out of the study. We were unable to follow-up 12 patients who completed group therapy (6 cases moved out of the region, 4 finished treatment, and 2 dropped out from outpatient clinic) and seven patients who dropped out of group therapy (all 7 patients dropped out from outpatient clinics). The completer group had a significantly higher number of patients who had a good prognosis (χ^2^(1) = 20.4, *p* < 0.01), and the dropout group had more patients who had a poor prognosis (*p* < 0.05).

**Table 3:** Outcomes of completers and dropouts

	Completers (*N* = 36)	Dropouts (*N* = 10)	*p*
Good	30^a^	0	<0.01
No change	5	6	NS
Poor	1	4	<0.05

^a^There were significantly more patients with good outcome in the completers group

We investigated the prognosis (good or poor) of the completer group and the dropout group 1, 5, and 10 years later. In the completer group: 1 year later, 22 cases (25%) were good and 3 (8.3%) were poor; 5 years later, 30 cases (83.3%) were good and 2 cases (5.6%) were poor; 10 years later, 30 cases (83.3%) were good and 2 cases (5.6%) were poor. In the dropout group: 1 year later, 1 case (10%) was good and 2 (20%) were poor; 5 years later: 0 cases (0%) were good and 4 cases (40%) were poor; 10 years later: 0 cases (0%) were good and 4 cases (40%) were poor.

## Discussion

As reported by Fairburn [10, 11], eating disorders, i.e., AN, BN, BED, and those of incomplete type, all have a common core psychopathology. We agree with Fairburn that transdiagnostic CBT (CBT-E) is appropriate for all eating disorders. Thus, we conducted a CBT intervention in a group setting for all types of eating disorders. Fairburn’s CBT-E manual is written for use with individuals, and not for group intervention. Individual therapy has merit, in that we can intervene in response to the situation of the individual patient, and it can be used for patients who tend to avoid group therapy. However, for other patients, group therapy is cost-effective and efficacious due to the social experience and the expectation of approval within the group. Therefore, we conducted group therapy to supplement short-term outpatient treatment. We devised a therapeutic method for group therapy based on the CBT-E protocol of Fairburn.

After intervention, eating attitudes, depressive mood, and emotional response to stress reduced, and self-esteem increased. Through awareness of the change, the participants seemed to have recovered self-esteem and were motivated to continue treatment. In our previous report [33], depression, anger, and confusion were more marked in women with eating disorders than in healthy women. Several reports have indicated that depressive symptoms predict treatment outcome [34, 35], and can indicate the risk for suicide. It is important to reduce depressive symptoms and to prevent suicide. The CBT intervention used in this study improved depressive symptoms.

The dropout rate from eating disorder treatment is said to be high because of the psychopathology involved in these disorders, such as denial of the severity of the disorder by the patient and treatment resistance [21]. Bandini et al. reported that the dropout rate of outpatients from CBT was 33% for AN-BP, 27% for AN-R, 25% for BN, and 21% for EDNOS [36]. Several factors, such as low patient engagement, purging episodes, restrictive eating, use of several weight control practices, and psychiatric co-morbidity affect dropout rates [37]. However, others have reported that purging does not influence the dropout rate [38], and instead that low depression scores [39], divorce of parents [39, 40], a tendency to repress anger [41], and self-efficacy [42] influence dropout. In this study, the dropout rate was 33.3% for AN-BP, 27.8% for AN-R, 25% for BN-P, and 22.2% for BN-NP. We had a small sample size and so significant differences between each type of eating disorder were not found, although the presence of purging was frequent in those who did not complete CBT. We found that in the dropout group, compared to the completer group, there was higher anxiety and anger, not depression. Thus, it is important to tackle resistance to, and anxiety towards, treatment, and to conduct the introduction to treatment carefully.

Regarding the 10-year outcomes, the completer group had a significantly higher number of patients with a good prognosis, and the dropout group had a significantly higher number of patients with a poor prognosis. We could track only 10 of 17 patients who dropped out from therapy. The 7 patients whose prognosis could not be followed all dropped out during the outpatient consultation phase. This, it is likely that the prognosis of these 7 cases was poor. Furthermore, the 5-year prognosis was similar to the 10-year prognosis; therefore, we speculate that it is possible to estimate the long-term prognosis by prognosis after 5 years. The therapy was a short-term intervention; thus, this therapy alone did not influence the long-term outcome. However, since there were fewer dropouts from outpatient clinics among patients who completed the group therapy, it is possible that the group therapy experience had a positive effect on subsequent treatment continuity and prognosis. It seems that the patients continued practicing the skills and methods learned from group therapy, and reconfirmed these skills in the outpatient consultations.

People with eating disorders experience social impairments [43]. Difficulties in interpersonal relationships have been reported as potential factors in the development and maintenance of body image disturbances and altered eating behaviors [44]. Group therapy gives patients an opportunity to form peer relationships, and helps them to develop communication and socialization skills. Furthermore, in the group therapy sessions, the patients develop self-awareness by listening to other members of the group and develop interpersonal relationships. If the patients successfully work together in the group setting, they derive benefits, such as the prevention of feelings of isolation and the mutual recognition of increased self-esteem. People with eating disorders generally have low self-esteem [1, 45–47], and improving self-esteem is one of the main targets of treatments for eating disorders [48, 49]. Interventions that improve self-esteem help in recovery from eating disorders. Considering this research, many patients who completed the group CBT continued outpatient treatment and showed a good prognosis. They showed improvement in depressive symptoms and recovery of self-esteem. This effect may be temporary, but the experience of group therapy may have provided hope and had a positive effect on continuation of treatment. Many Japanese patients experience difficulties when speaking to others with the same illness and talking about themselves. Therefore, it is valuable to provide a place to gather as a group, and opportunities for group discussion. In addition, self-assertion sessions are advisable because Japanese patients are often passive and willing to adapt to others without stating their own opinions. Furthermore, Japanese individuals often have a strong sense of guilt if they suffer from eating disorders. It seemed that the motivation to continue treatment was enhanced by the mutual support in the group, and by completing group therapy, which led to a sense of accomplishment and confidence.

A limitation of this study is its small sample size. It is difficult to confidently state that the non-completers had poor prognosis, because they were few in number. Further studies are required to replicate these findings with a control group, preferably using an RCT.

Another limitation of our study is that we could not follow up 26% of the subjects for the full 10 years. In retrospect, a more robust method of follow-up could have been determined at the start of study. In addition, social adaptation was assessed using the GAF; however, the DSM-5 proposes that one should use the WHO Disability Assessment Schedule (WHODAS). WHODAS is a multifaceted assessment of living functions from 6 areas, and if it had been used, we might have appreciated better the patients’ living conditions.

It appeared that the participants of this study had a considerable number of developmental disorders including autism spectrum disorder. However, for the diagnosis of developmental disorders, detailed childhood information is necessary, and since there are limitations to retrospective investigation, it was not reported in this study.

Finally, it is thought that not only short-term interventions, but also additional CBT sessions, may be more effective. In future, we would like to test this hypothesis.

## Conclusions

Depressive symptoms, emotion-oriented coping, and self-esteem were improved after the integrated cognitive-behavioral therapy in a group setting. Many patients who completed group therapy had a good 10-year prognosis.

## Abbreviations

AN: Anorexia nervosa
AN-BP: Anorexia nervosa binge-eating/purging type
AN-R: Anorexia nervosa restricting type
BED: Binge-eating disorder
BMI: Body mass index
BN: Bulimia nervosa
CBT: Cognitive behavioral therapy
CBT-E: Enhanced cognitive behavioral therapy
CISS: Coping Inventory for Stressful Situations
DSM: Diagnostic and Statistical Manual of Mental Disorders
EAT-26: Eating Attitudes Test-26
EDNOS: Eating disorder not otherwise specified
GAF: Global Assessment of Functioning
OSFED: Other specified feeding and eating disorders
POMS: Profile of Mood States
RSES: Rosenthal’s Self Esteem Scale
WHODAS: WHO Disability Assessment Schedule
